# Influence of individual quadriceps and hamstrings muscle architecture and quality on knee adduction and flexion moment in gait

**DOI:** 10.1038/s41598-023-47376-2

**Published:** 2023-11-24

**Authors:** Jiyoung Jeong, Dai-Hyuk Choi, Choongsoo S. Shin

**Affiliations:** 1 Department of Mechanical Engineering, Sogang University, 35 Baekbeom-ro, Mapo-gu, Seoul, 04107 Republic of Korea; 2 Department of Physical Education, Graduate School of Education, Sogang University, 35 Baekbeom-ro, Mapo-gu, Seoul, 04107 Republic of Korea

**Keywords:** Musculoskeletal system, Risk factors

## Abstract

The purpose of this study was to investigate the relationship between muscular parameters of quadriceps/hamstrings and knee joint kinetics in gait. Muscle architecture (thickness, pennation angle, and fascicle length), and quality (echo intensity) of individual quadriceps and hamstrings of 30 healthy participants (16 males and 14 females) was measured using ultrasound. Peak knee flexion moment (KFM), KFM impulse, peak knee adduction moment (KAM), and KAM impulse during walking were obtained at preferred speed. Pearson’s correlation coefficient and multiple regression analyses were performed at significance level of 0.05, and Cohen’s *f*^2^ values were calculated to examine the effect sizes of multiple regression. The hamstring-to-quadriceps muscle thickness ratio (r = 0.373) and semitendinosus echo intensity (r =  − 0.371) were predictors of first peak KFM (R^2^ = 0.294, *P* = 0.009, *f*^2^ = 0.42), whereas only vastus medialis (VM) echo intensity was a significant predictor of second peak KFM (r = 0.517, R^2^ = 0.267, *P* = 0.003, *f*^2^ = 0.36). Only the VM thickness was the predictor of first (r = 0.504, R^2^ = 0.254, *P* = 0.005, *f*^2^ = 0.34) and second peak KAM (r = 0.581, R^2^ = 0.337, *P* = 0.001, *f*^2^ = 0.51), and KAM impulse (r = 0.693, R^2^ = 0.480, *P* < 0.001, *f*^2^ = 0.92). In conclusion, the greater hamstring-to-quadriceps muscle thickness ratio and the muscle architecture and quality of medial quadriceps/hamstring play an important role in KFM and KAM, and may have implications in knee osteoarthritis.

## Introduction

Clinical gait analyses provide quantitative information regarding gait pattern alterations, thereby aiding the understanding of gait abnormalities and clinical decision-making for a patient’s functional limitation due to disorder and its follow-up evaluation^1^. In particular, gait patterns associated with knee osteoarthritis (OA) have been broadly described across the literature^2^. The external knee adduction moment (KAM) and impulse are commonly considered a surrogate measure of dynamic loading over the medial knee compartment^3^. Since these parameters are associated with the increased risk of medial cartilage damage possibly leading to medial knee OA, they are known as biomechanical risk factor for the development and progression of OA^3,4^. In addition, combination of the KAM and the knee flexion moment (KFM) could be a better predictor of knee loading than the KAM alone, because the KFM also plays a role in medial knee joint loading^5^. Therefore, investigating the factors that contribute to reduction of KAM and KFM can help design intervention programs to mitigate the disease presence or process for the individuals at higher risk of knee OA.

Previous studies have demonstrated that modifiable factors, such as gait speed, stride length, and foot progression angle, can contribute to gait modification, thereby altering the medial knee joint load^6^. These factors, as well as the joint biomechanics, are associated with the muscular parameters of the lower extremity^7,8^. Specifically, muscle thickness of lateral thigh muscle was positively correlated with gait speed and its echo intensity, which is indicative of muscle quality by representing the quantity of intramuscular fibrous and adipose tissue in muscle, was negatively correlated with step length^7^. Kumar et al.^8^ also reported that greater medial-to-lateral quadriceps anatomical cross-sectional area ratio was associated with greater knee adduction moment in gait. Although the quadriceps and hamstrings are important contributors to knee joint loading during walking^8,9^, no studies have investigated the relationships between the their individual muscular parameters and the knee biomechanics in gait. In addition, a previous study reported that the greater muscle thickness of medial thigh muscles relative to lateral thigh muscles was associated with a lower knee valgus moment (i.e. knee abduction moment) during landing^10^, but the relationship between the other muscle architecture and/or quality of the individual quadriceps and hamstrings and the knee joint moments during walking remains unclear.

This study aims to examine the relationship between the muscular parameters of individual quadriceps and hamstrings and knee joint kinetics in gait. It has been hypothesized that the greater muscle thickness (i.e. better muscle strength) and/or lower echo intensity (i.e. better muscle quality) of some individual quadriceps and hamstrings would result in lower KAM and KFM and/or moment impulse during walking. Specifically, the muscular parameters of the medial quadriceps and hamstrings would be associated with the KAM, while the hamstring-to-quadriceps (H:Q) muscle thickness ratio would be associated with the KFM. In addition, the muscular parameters of the quadriceps and hamstrings would be associated with the gait spatiotemporal parameters such as stride length and walking speed.

## Results

### Correlations with knee joint kinetics in sagittal plane

**Figure 1:**
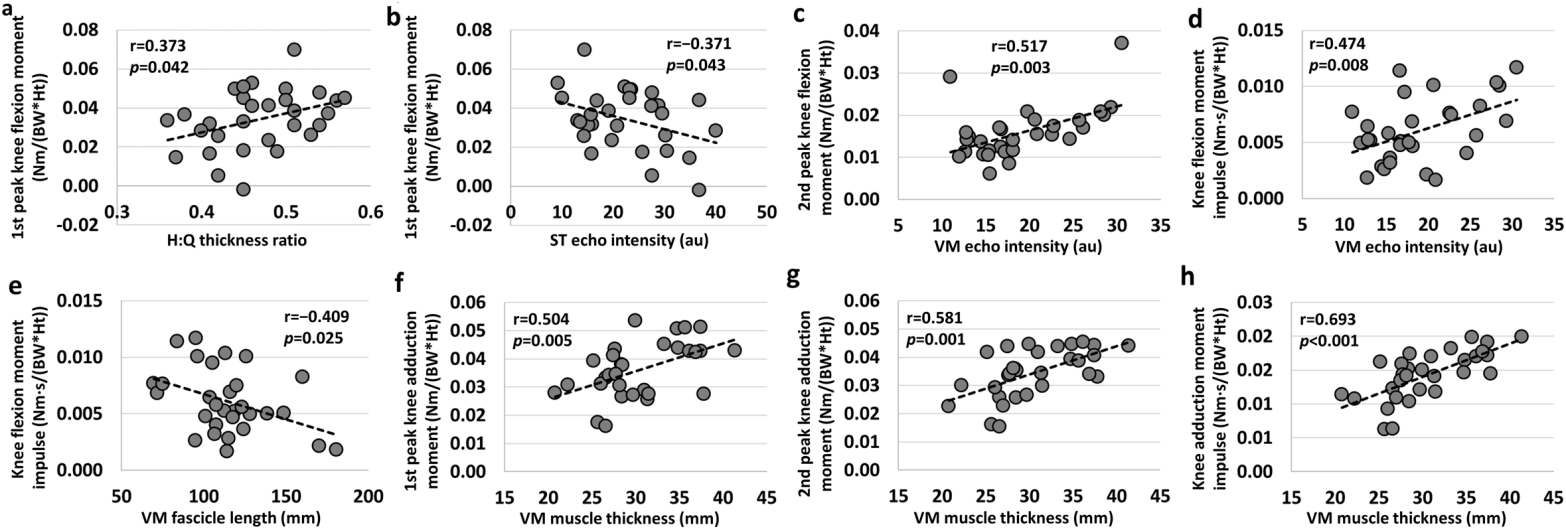
Relationship between the muscular parameters of individual quadriceps/hamstrings and the knee joint kinetics in sagittal and frontal planes during walking. The 1st peak knee flexion moment was correlated positively with (**a**) the hamstring-to-quadriceps (H:Q) thickness ratio and negatively with (**b**) the semitendinosus (ST) echo intensity. The 2nd peak knee flexion moment was positively correlated with (**c**) the vastus medialis (VM) echo intensity. The knee flexion moment impulse was correlated positively with (**d**) the VM echo intensity and negatively with (**e**) the VM fascicle length. Both the 1st knee adduction moment and the 2nd knee adduction moment were positively correlated with the VM muscle thickness (**f**,**g**). The knee adduction moment impulse was positively correlated with (**h**) the VM muscle thickness. Corresponding muscular parameters were derived from stepwise multiple regression analysis.

Knee joint kinetics during walking demonstrated significant correlation with the physiological properties of individual quadriceps and hamstrings. The first peak KFM was positively associated with the H:Q thickness ratio (r = 0.373, *P* = 0.042; Fig. 1a) but also negatively associated with the VM muscle thickness (r =  − 0.365, *P* = 0.048), VM fascicle length (r =  − 0.371, *P* = 0.043), and ST echo intensity (r =  − 0.371, *P* = 0.043; Fig. 1b) (Supplementary Table S1). Multiple regression analysis revealed that the H:Q thickness ratio and ST echo intensity were predictors of the first peak KFM (R^2^ = 0.294, *P* = 0.009, *f*^2^ = 0.42; Table 1). Only the VM echo intensity was a significant predictor of the second peak KFM (R^2^ = 0.267, P = 0.003, *f*^2^ = 0.36; Table 1, Fig. 1c). Additionally, the VM echo intensity and fascicle length were predictors for the KFM impulse (R^2^ = 0.386, P = 0.001, *f*^2^ = 0.63; Table 1). The VM echo intensity was positively associated with the KFM impulse (r = 0.474, *P* = 0.008; Fig. 1d), whereas the VM fascicle length was negatively associated with it (r =  − 0.409, *P* = 0.025; Fig. 1e) (Supplementary Table S1).

**Table 1:** Multiple linear regression equations.

Dependent variable	Regression equation	R^2^	*P*	*f*^2^
1st peak FM	(0.107*HQratio) + (− 0.001*EIST) + 0.001	0.294	0.009	0.42
2nd peak FM	(0.001*EIVM) + 0.005	0.267	0.003	0.36
FM impulse	(0.000*EIVM) + (− 0.000*FLVM) + 0.007	0.386	0.001	0.63
1st peak AM	(0.001*MTVM) + 0.006	0.254	0.005	0.34
2nd peak AM	(0.001*MTVM) + 0.004	0.337	0.001	0.51
AM impulse	(0.001*MTVM) − 0.001	0.480	0.000	0.92
Speed	(− 0.005*EIST) + (− 0.024*PAVI) + (0.019*PAVM) + 1.402	0.443	0.001	0.80
Stride	(− 0.026*MTBF) + 2.223	0.182	0.019	0.22

*AM* adduction moment; *BF* biceps femoris; *EI* echo intensity; *FL* fascicle length; *FM* flexion moment; *HQratio* hamstring-to-quadriceps thickness ratio; *MT* muscle thickness; *PA* pannation angle; *VI* vastus intermedius; *VM* vastus medialis; *ST* semitendinosus.

### Correlations with knee joint kinetics in frontal plane

Several muscular parameters of individual quadriceps and hamstrings were significantly associated with the peak KAMs and moment impulse (Supplementary Table S1); however, the VM thickness was the only predictor of the first peak KAM (R^2^ = 0.254, *P* = 0.005, *f*^2^ = 0.34; Fig. 1f), the second peak KAM (R^2^ = 0.337, *P* = 0.001, *f*^2^ = 0.51; Fig. 1g), and the KAM impulse (R^2^ = 0.480, *P* < 0.001, *f*^2^ = 0.92; Fig. 1h), respectively (Table 1).

### Correlations with gait spatiotemporal parameters

The gait speed and stride length were also found to be significantly associated with muscular parameters of both the quadriceps and hamstrings (Supplementary Table S1). The ST echo intensity (r =  − 0.436, *P* = 0.016), VI pennation angle (r =  − 0.396, *P* = 0.030), and VM pennation angle (r = 0.367, *P* = 0.046) were predictors of the gait speed (R^2^ = 0.443, *P* = 0.001, *f*^2^ = 0.80; Table 1). The stride length was found to be negatively associated with the BF muscle thickness (r =  − 0.427, *P* = 0.019) and the VI pennation angle (r =  − 0.374, *P* = 0.042); however, only the BF thickness was a significant predictor of the stride length (R^2^ = 0.182, P = 0.019, *f*^2^ = 0.22; Table 1).

## Discussion

The primary finding of this study was that the muscular parameters of the individual quadriceps and hamstring determine the correlative properties of knee joint moments and the gait spatiotemporal parameters. These linear relationships can be used to estimate the gait parameters from the muscle architecture and quality; thus, it is important to consider the ultrasound image assessment of the individual quadriceps and hamstrings when predicting the knee joint kinetics in both the sagittal and frontal planes or the gait spatiotemporal parameters.

A significant correlation between the sagittal plane knee kinetics and the muscular parameters of the individual quadriceps and hamstrings was found. Specifically, the peak KFM in the first half of stance was positively associated with the H:Q muscle thickness ratio but negatively associated with the ST echo intensity, whereas the second peak KFM was positively associated with the VM echo intensity. This implies that the lower muscle quality of VM and the higher quality of ST, as well as the greater muscle thickness of the hamstrings compared to the quadriceps, can generate a greater KFM, because a higher echo intensity is indicative of lower muscle quality. A previous study investigating the relationship between quadriceps strength and knee joint mechanics during walking in individuals with knee OA reported that the strength of the quadriceps was significantly positively correlated with peak knee flexion angle, whereas it was not associated with peak KFM^11^. Given that the relative hamstring muscle thickness to the quadriceps and the medial thigh muscle quality (i.e., echo intensity of VM and ST) demonstrated predictive relationships with peak KFM, the hamstring muscle thickness compared to the quadriceps and the muscle quality of the medial quadriceps and hamstrings appeared to play a dominant role in controlling the peak KFMs during walking; thus, these specific parameters need to be considered to estimate the sagittal knee joint kinetics during walking.

In this study, only the VM thickness, which was positively associated with knee kinetics in the frontal plane during walking, could predict both the first and second peak KAMs and moment impulse. This result was similar to that by Zhang et al. who reported that the lateral muscles of the lower extremity generated knee abduction moment, while the medial muscles generated KAM^12^. In addition, the low ratio of medial to lateral vastii muscle recruitment can cause the lateral part of the knee joint to be compressed and the medial joint to be opened, causing knee valgus during a reproducible maneuver that mimics the high anterior cruciate ligament injury risk position^13^, and a negative correlation was found between the VM to VL activation ratio and the knee valgus^14^. These vastii muscles act as a knee joint stabilizer during weight-bearing. Furthermore, the KAM during walking was not correlated with knee extensor strength^15^. Taken together, the medial quadriceps appeared to play an important role in attenuating the frontal plane loads by influencing the KAM, thereby contributing to the knee joint stability during walking.

Previously, KAM and impulse were generally identified as predictors of the presence and progression of medial compartment tibiofemoral joint OA^3^. Considering the risk of patellofemoral joint injury, higher KFM and KFM impulse in the second half of the gait stance contribute to the progression of patellofemoral joint OA^16^. Walter et al.^17^ reported that reductions in the peak KAM does not always guarantee corresponding reductions in medial knee contact force owing to the corresponding increase in peak KFM; hence, the KFM is also important controlling factor for the medial knee OA. According to a recent meta-analysis, the resistance strength training of the lower extremity has no significant effects on the KAM in patients with knee OA^18^. In addition, the neuromuscular exercise and quadriceps strengthening do not change the KAM and KFM^19^. However, a previous study also reported that a greater VL cross-sectional area relative to VM is associated with a decreased risk of knee OA^20^ and strengthening the lateral muscle knee chain can decrease the load on the medial compartment^21^; hence, specific intervention programs by selectively strengthening the individual quadriceps and hamstrings seemed to be beneficial in reducing the knee OA risk. The muscle thickness, the fascicle length and the pennation angle increased and the echo intensity reduced following training interventions^22–24^. Based on the current study results, strengthening VM can be conflicting in terms of knee joint loading because improved VM was associated with the lower peak KFM and KFM impulse, but greater peak KAMs and KAM impulse at the same time. Therefore, our study results could support the necessity of a new gait-related functional intervention program that aims to improve the quadriceps to hamstring ratio but, simultaneously, does not intensively strengthen the medial thigh muscles. Future studies are needed to determine the optimum medial–lateral quadriceps and hamstring strength ratio and to evaluate whether modifying the individual thigh muscle architecture and quality can change knee joint kinetics in gait, thereby reducing the knee OA risk.

In this study, the muscular parameters of individual quadriceps and hamstrings help explain the spatiotemporal gait parameters. Many previous studies investigating the effects of gait speed on gait features have demonstrated that the amplitude of joint kinematics and kinetics changes in response to the varying gait speed^25^. Accordingly, the prediction methods for gait kinematics and kinetics using gait speed have been suggested. In this study, gait speed and stride length demonstrated a predictive dependency on the muscular parameters of individual quadriceps and hamstrings; thus, the muscle architecture and quality of thigh muscles can be used as predictors of knee joint kinetics in self-selected gait strategies.

Although our results showed that the muscle architecture and quality of individual quadriceps and hamstring were associated with knee joint kinetics in both the sagittal and frontal planes during walking and gait spatiotemporal parameters, it is unclear how these muscular parameters determine the knee joint kinetics in gait. However, we do know from previous studies that force and/or moment generating capacity changes with the muscle force–length and force–velocity properties^26,27^. Muscle architecture can influence these force–length and force–velocity relationships. Specifically, muscles with longer fascicle generates the greater force at all muscle velocities^28^. It has been also reported that the physiological cross-sectional area (PCSA), which is approximated from muscle volume, fascicle length, and pennation angle, was the primary reason of the magnitude of the peak joint moment^26^. Therefore, it is plausible that the muscular force–velocity and force–length properties and the muscle PCSA, which can be determined by muscle architecture, contributes to the gait performance.

There are some limitations to be considered when interpreting the study results because other factors could affect knee kinetics during walking. First, only young and healthy participants with no history of musculoskeletal injury were included in this study. As previous studies reported age-related differences in gait kinematics and kinetics^29^, the results cannot be generalized to the overall population, including the elderly, children, and/or injured people. Thus, a future study should consider expanding the research participants to determine whether the observed relationships occur across different age groups as well. Additionally, muscular parameters of non-knee-spanning muscles such as the gluteal muscles were not considered, although these parameters contribute to knee joint kinetics during the dynamic movement. Therefore, further investigations are recommended for a deeper understanding of the effect of the lower extremity muscle parameters on joint kinetics/kinematics during walking. Finally, the muscle architecture and quality were measured at a single site in this study, so it seemed difficult to fully reflect the muscle volume. Although the quantification of muscle volume using MRI has been recognized as a gold standard and might be more predictive of joint kinetics, measuring muscular parameters using ultrasound can be more useful as a relatively simple and less expensive means of predicting the knee joint moment in gait.

In conclusion, the muscular parameters of the individual quadriceps and hamstrings demonstrated a significant correlation with the knee joint kinetics and spatiotemporal parameters during walking, thereby estimating the gait features in young and healthy populations. This result implies that the relative muscle thickness of the hamstrings to the quadriceps as well as the medial quadriceps and hamstrings (i.e., VM and ST) muscle architecture and quality play an important role in KFM and KAM and may have implications in knee OA.

## Methods

### Subjects

An a priori power analysis using G*power software version 3.1.9.4, based on the pilot data which showed a correlation coefficient of 0.7 between the vastus medialis (VM) muscle thickness and the KAM impulse during walking revealed that obtaining a power of 0.80 with an alpha level of 0.05 required a sample size of at least 13 participants. Thirty recreationally active males (n = 16, age: 22.7 ± 1.9 yrs, height: 174.5 ± 2.6 cm, mass: 69.0 ± 6.3 kg, body mass index (BMI): 22.2 ± 2.3 kg/m^2^) and females (n = 14, age: 20.9 ± 2.9 yrs, height: 161.0 ± 5.1 cm, mass: 54.2 ± 7.9 kg, BMI: 21.7 ± 2.9 kg/m^2^) participated in this study. Participants were excluded if they had a lower extremity injury experience in the past six months that prevented participation in physical activity for more than 2 weeks or if they had a history of knee injuries or other musculoskeletal injuries requiring surgery. This study was approved by the ethics committee of Sogang University (SGUIRB-A-1808-44). Prior to participation, all participants signed an informed consent form. All methods in this study were conducted according to the relevant guidelines and regulation of the Declaration of Helsinki.

### Procedures

**Figure 2:**
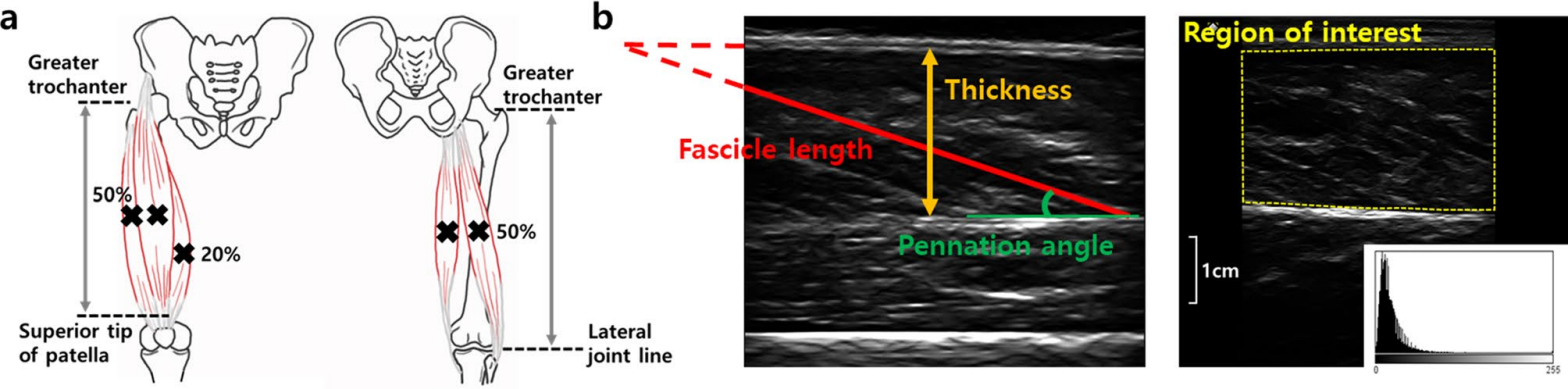
(**a**) Ultrasound imaging assessment for individual quadriceps and hamstrings (**b**) Example for measuring the muscular parameters.

Ultrasound images of the rectus femoris (RF), vastus lateralis (VL), VM, vastus intermedius (VI), biceps femoris (BF), and semitendinosus (ST) on the dominant limb were obtained using B-mode ultrasound (MicrUs EXT-1H, Telemed Ultrasound Medical System, Milano, Italy) with a linear array probe (10 MHz). Ultrasound settings, including gain (60 dB), depth (70 mm), and frequency (10 MHz), were set prior to testing. Sufficient ultrasound gel was used to minimize muscle compression of the probe head. With the participant lying supine and both feet fixed to prevent hip external rotation, the thickness of VM was measured at 20% of the distance between superior tip of the patella and the anterior superior iliac spine, and RF, VI, and VL at 50% of this distance above the patella (Fig. 2a)^30^. All ultrasound images of BF and ST were scanned in the sagittal plane at 50% of the distance between the greater trochanter and the lateral joint line of the knee in the prone position with the lower limb extended and relaxed (Fig. 2a)^31^. For each session, two ultrasound images were captured, and the mean was calculated for each muscular parameter.

A three-dimensional motion capture system equipped with ten infrared cameras (nine Eagle, and one Raptor; Motion Analysis Corp., Santa Rosa, CA, USA) was used to record the motion of the knee joint at a sampling rate of 400 Hz. Reflective markers (i.e., Ф12.5 mm spheres) were placed on the anatomical bony landmarks: left and right acromion, sternum, right scapula, bilateral anterior superior iliac spines, sacrum, greater trochanter, midpoint of the femur, lateral and medial epicondyles of the femur, lateral and medial plateau of the tibia, midpoint of the tibia, lateral and medial malleolus, calcaneus, and the first and the fifth metatarsal heads. A force plate (9260AA6; Kistler, Winterthur, Switzerland) embedded in the floor was used at a sampling rate of 1200 Hz, synchronized with the motion capture system to calculate the joint moments.

All participants walked at a self-selected pace and were instructed to step on a force plate with the dominant limb. Leg dominance was defined as the preferred leg for kicking a ball. If there was no clean-foot contact on the force plate and/or the participants failed to maintain the walking speed, then the corresponding trials were discarded. To eliminate the effect of footwear, all participants wore the same type of running shoes: Nike Downshifter 8 (NIKE Inc., Beaverton, OR, USA).

All ultrasound images were analyzed using ImageJ software (Version 1.51 k, National Institutes of Health, Bethesda, MD) to measure the muscular parameters (i.e., muscle thickness, pennation angle, fascicle length, and echo intensity) of the RF, VI, VL, VM, BF, and ST (Fig. 2b). The muscle thickness was defined as the distance between the superficial and deep fasciae of each muscle. The pennation angle was determined as the angle between the deep aponeurosis and fascicle, and the fascicle length was determined as the length of the fascicular path between superficial and deep aponeurosis. When the ends of the fascicles were outside the probe field-of-view, the length of the missing portion of the fascicle was estimated by extrapolating linearly both the fascicular path and the aponeurosis. In addition, the echo intensity was determined as an indicator of skeletal muscle quality by gray-scale analysis using the histogram function in ImageJ. Regions of interest including as much of each muscle as possible but avoiding the surrounding fascia were selected. The mean echo intensity of the regions was expressed as a value between 0 (black) and 255 (white). The same investigator that performed the scans also performed the stored image measurements. The H:Q thickness ratio was calculated as the sum of muscle thickness of knee flexors (i.e. BF and ST) divided by the sum of muscle thickness of knee extensors (i.e. RF, VI, VM, and VL), and the medial-to-lateral thigh muscle thickness ratio was calculated as the sum of muscle thickness of VM and ST divided by the sum of muscle thickness of VL and BF.

Each gait cycle was defined as the period between the initial heel strike and subsequent heel strike of the same foot. The initial heel strike of walking was identified by determining the first frame at which the vertical ground reaction force exceeded 20 N. The measured kinetic data were filtered using a zero-lag fourth-order Butterworth low-pass filter at a cutoff frequency of 20 Hz. The joint moments were calculated by solving an inverse dynamics problem using Newton–Euler equations. Joint moments were expressed as external moments and normalized to the body weight and height of each participant. The joint moment impulse was calculated as the integral of the moment with respect to time. Peak KAM and KFM, moment impulses, stride length (normalized to leg length), and walking speed were sampled to determine whether thigh muscular parameters can determine gait kinetics.

### Statistical analyses

The two successful trials were averaged individually first and then averaged to generate the group mean values and the standard deviation. The Kolmogorov–Smirnov test was used to check the data distribution normality. The results revealed that all recorded data were normally distributed (*P* > 0.05). A high degree of reliability was demonstrated using a two-way mixed model (intraclass correlation coefficient; ICC_3,1_) to assess the agreement in all muscular parameters between the data measured across two trials on separate days by one rater (ICC_3,1_ ≥ 0.830). Pearson’s correlation coefficient was used to determine the relationship between the muscular parameters of individual quadriceps and hamstrings and the knee joint kinetic variables, gait speed, and stride length. When significant correlations were found, stepwise multiple regression analysis was performed to investigate the muscular parameters that could explain the knee joint biomechanics and gait spatiotemporal parameters. Cohen’s *f*^2^ values were calculated to examine the effect sizes of multiple regression: *f*^2^ of 0.02, 0.15, and 0.35 indicate a small, medium, and large effect size, respectively. All statistical analyses were performed using MATLAB version R2018a (MathWorks Inc., Natick, MA, USA).

### Supplementary Information

Supplementary Table S1.

## Data Availability

The datasets generated during and/or analyzed during the current study are available from the corresponding author on reasonable request.
